# P-268. Missed Opportunities and Factors Associated with Late Diagnosis of HIV Infection in the Era of Rapid Testing

**DOI:** 10.1093/ofid/ofaf695.489

**Published:** 2026-01-11

**Authors:** Federico Daniel Cardozo, Sofia Frankel, Guillermo Alberto Viloria, Isabel Pastor, Marina Alonso Serena, Belen Zorz, Jimena Lopez Pineiro, Iael Altclas, Javier Toibaro, Lucas Saucedo, Elena Beatriz Aldea Cieza, Angel Parlante, Mariana Angelica Kundro, Marcelo H Losso

**Affiliations:** Hospital General de Agudos Jose Maria Ramos Mejia, Ciudad Autonoma de Buenos Aires, Ciudad Autonoma de Buenos Aires, Argentina; Hospital General de Agudos Jose Maria Ramos Mejia, Ciudad Autonoma de Buenos Aires, Ciudad Autonoma de Buenos Aires, Argentina; Hospital General de Agudos José María Ramos Mejía, Ciudad Autonoma de Buenos Aires, Ciudad Autonoma de Buenos Aires, Argentina; Hospital JM Ramos Mejía, Buenos Aires, Ciudad Autonoma de Buenos Aires, Argentina; Hospital JM Ramos Mejía, Buenos Aires, Ciudad Autonoma de Buenos Aires, Argentina; Hospital General de Agudos Ramos Mejia, ciudad autonoma de buenos aires, Ciudad Autonoma de Buenos Aires, Argentina; Hospital General de Agudos Ramos Mejia, ciudad autonoma de buenos aires, Ciudad Autonoma de Buenos Aires, Argentina; Hospital Donación Francisco Santojanni, Ciudad Autonoma de Buenos Aires, Ciudad Autonoma de Buenos Aires, Argentina; Hospital General de Agudos Ramos Mejia, ciudad autonoma de buenos aires, Ciudad Autonoma de Buenos Aires, Argentina; Hospital General de Agudos Ramos Mejia, ciudad autonoma de buenos aires, Ciudad Autonoma de Buenos Aires, Argentina; Hospital General de Agudos Ramos Mejia, ciudad autonoma de buenos aires, Ciudad Autonoma de Buenos Aires, Argentina; Hospital General de Agudos Ramos Mejia, ciudad autonoma de buenos aires, Ciudad Autonoma de Buenos Aires, Argentina; Hospital General de Agudos José María Ramos Mejía, Ciudad Autonoma de Buenos Aires, Ciudad Autonoma de Buenos Aires, Argentina; Hospital JM Ramos Mejía, Buenos Aires, Ciudad Autonoma de Buenos Aires, Argentina

## Abstract

**Background:**

Late diagnosis (LD) of HIV infection (CD4+ < 350 cel/μl or AIDS defining condition) remains a challenge in our setting despite efforts to increase HIV testing strategies and widespread availability of rapid testing. Our aims were to assess the factors associated with LD in a cohort of patients with a new HIV diagnosis and to evaluate missed opportunities and barriers perceived by patients to timely HIV testing.

**Table 1.**
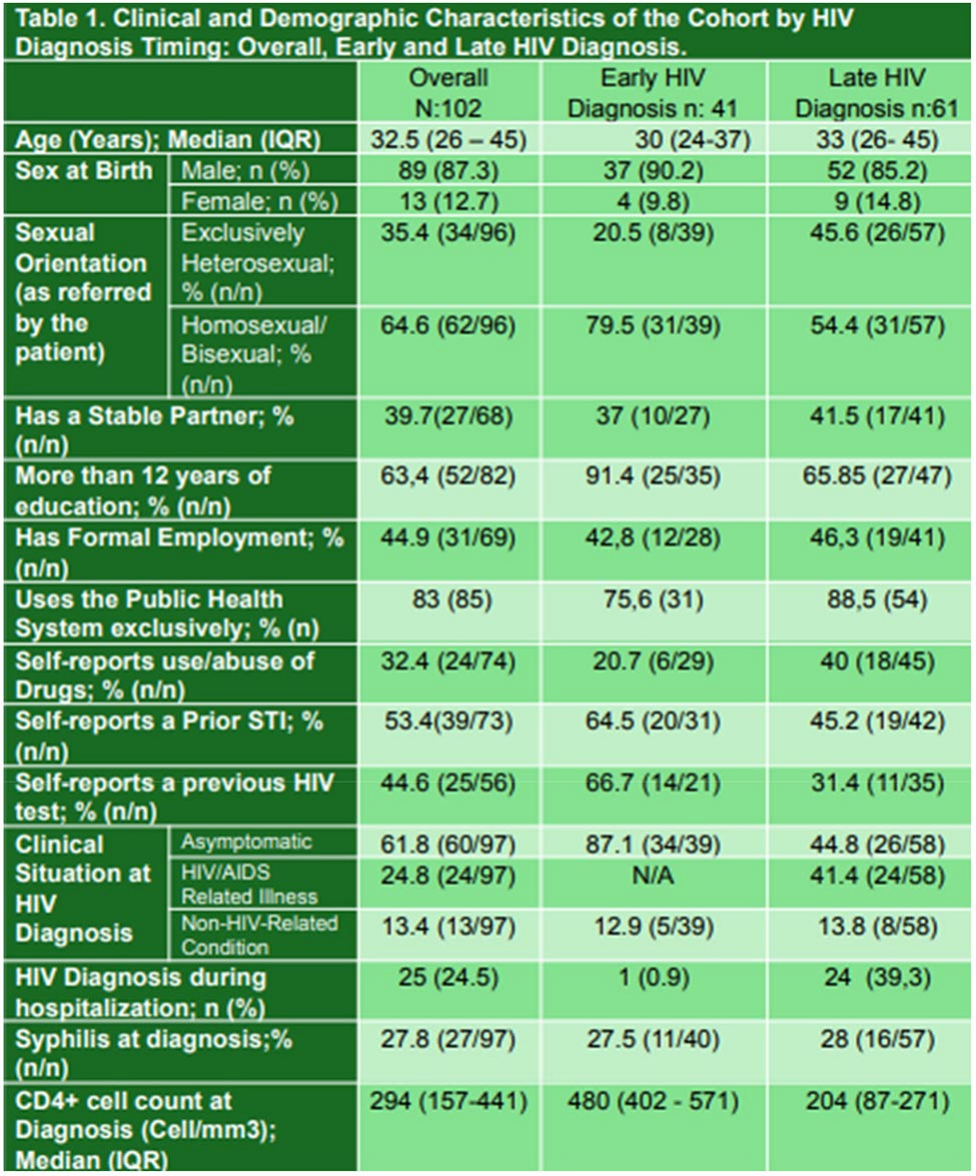
Clinical and Demographic Characteristics of the Cohort by HIV Diagnosis Timing: Overall, Early and Late HIV Diagnosis.

**Figure 1.**
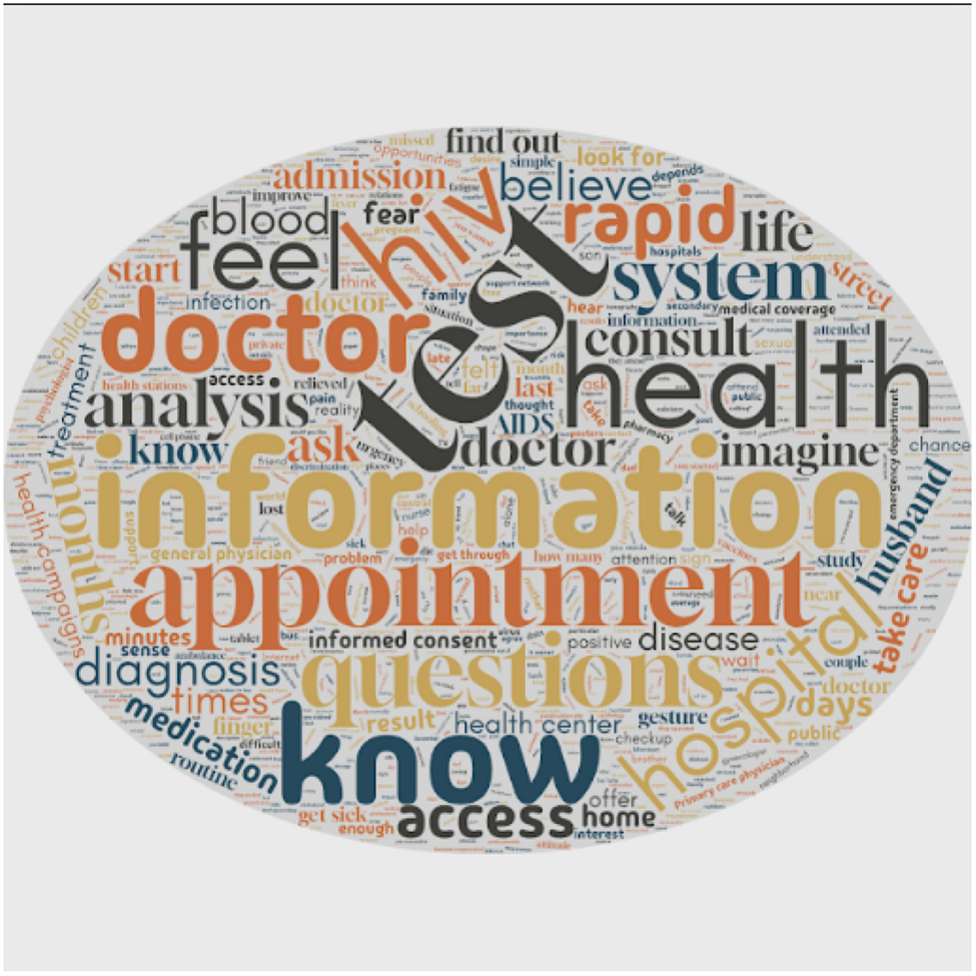
Word Cloud: Late Diagnosis Participants Key Terms

**Methods:**

Prospective, observational and analytical cohort study that included a qualitative analysis of patients with LD through in-depth interviews. All adults with a recent HIV diagnosis performed in a referral hospital in Bs Aires, Argentina from October 2023 to January 2025 were included.

**Results:**

102 patients were enrolled (Table 1), of whom 59.80% had LD criteria. The overall median CD4+ was 294 (IQR 157-441 cel/μl). As much as 36% of the subjects had ≥ 1 prior medical consultation in the previous year. 7 patients died during the study due to AIDS-related causes. 26.5% of the patients did not return for follow-up visits. 92% (51/55) of the subjects achieved virological suppresion at 6 months. The factors associated with LD were older age [OR: 1.07 (95% CI 1.02 to 1.10); p:0.002], and exclusively heterosexual relations [(OR: 2.61; 95% CI 1.07-6.33); p:0,03]. Sex (male vs female), years of education (≥ vs < 12), having formal employment, stable partner, drug use and history of STIs were not associated with a higher risk of LD in this cohort (p >0.05). The qualitative analysis highlighted that the majority of participants did not access routine health check-ups despite identifying nearby care centers, mainly due to self-perception of good health. Most of the individuals mentioned previous negative experiences in the health system, indicating that they had felt expelled by depersonalized care, and recognized missed opportunities for early diagnosis due to lack of offering the HIV test in previous consultations. Gaps were found in the dissemination of information about rapid HIV testing among most adult age groups.

**Conclusion:**

In our cohort, over half of the patients were diagnosed with late stage HIV infection despite previous interactions with the healthcare system, underscoring missed opportunities for testing. The need for broader and more targeted testing outreach strategies is stressed.

**Disclosures:**

All Authors: No reported disclosures

